# Reduced DNA Repair Capacity in Prostate Cancer Patients: A Phenotypic Approach Using the CometChip

**DOI:** 10.3390/cancers14133117

**Published:** 2022-06-25

**Authors:** Carmen Ortiz-Sánchez, Jarline Encarnación-Medina, Jong Y. Park, Natasha Moreno, Gilberto Ruiz-Deya, Jaime Matta

**Affiliations:** 1 Department of Basic Sciences, Ponce Research Institute, Ponce Health Sciences University, Ponce, PR 00716-2347, Puerto Rico; jencarnacion@psm.edu (J.E.-M.); jmatta@psm.edu (J.M.); 2 Department of Cancer Epidemiology, H. Lee Moffitt Cancer Center, Tampa, FL 33612, USA; jong.park@moffitt.org; 3 St. Luke’s Episcopal Hospital, Ponce, PR 00733, Puerto Rico; advancelaparoscopic@gmail.com (N.M.); gruiz@psm.edu (G.R.-D.); 4 Department of Surgery, Ponce Health Sciences University, Ponce, PR 00716-2347, Puerto Rico

**Keywords:** prostate cancer, DNA repair capacity, nucleotide excision repair, CometChip

## Abstract

**Simple Summary:**

Prostate cancer (PCa) is the most commonly diagnosed cancer type in Hispanic men in the US. Among Hispanics, Puerto Rican (PR) men show the highest PCa-specific mortality. Various studies have shown that having low DNA repair capacity (DRC) is a significant risk factor for cancer development. The aim of this study was to evaluate variations in DRC, through the nucleotide excision repair (NER) pathway, in PR men with PCa using the CometChip. Overall, PCa cases had lower DRC than controls. When PCa cases were stratified into aggressive and indolent, controls had higher DRC than both groups. The contributions of additional factors (i.e., age and prostate-specific antigen levels) to DRC were also considered. Our data suggest that DRC levels may have the potential to discriminate between aggressive and indolent cases. Our results represent an innovative step in the development of a blood-based screening test for PCa based on DRC levels.

**Abstract:**

Prostate cancer (PCa) accounts for 22% of the new cases diagnosed in Hispanic men in the US. Among Hispanics, Puerto Rican (PR) men show the highest PCa-specific mortality. Epidemiological studies using functional assays in lymphocytes have demonstrated that having low DRC is a significant risk factor for cancer development. The aim of this study was to evaluate variations in DRC in PR men with PCa. Lymphocytes were isolated from blood samples from PCa cases (*n* = 41) and controls (*n* = 14) recruited at a hospital setting. DRC levels through the nucleotide excision repair (NER) pathway were measured with the CometChip using UVC as a NER inductor. The mean DRC for controls and PCa cases were 20.66% (±7.96) and 8.41 (±4.88), respectively (*p* < 0.001). The relationship between DRC and tumor aggressiveness was also evaluated. Additional comparisons were performed to evaluate the contributions of age, anthropometric measurements, and prostate-specific antigen levels to the DRC. This is the first study to apply the CometChip in a clinical cancer study. Our results represent an innovative step in the development of a blood-based screening test for PCa based on DRC levels. Our data also suggest that DRC levels may have the potential to discriminate between aggressive and indolent cases.

## 1. Introduction

In 2022, approximately 268,490 new prostate cancer (PCa) cases will be diagnosed in the US according to the American Cancer Society. PCa will represent 14% of all new cancer cases diagnosed, and the most commonly diagnosed cancer in men [1]. It is estimated that, in 2022, around 34,500 PCa-related deaths will occur in the US. This makes PCa the second leading cause of cancer-related mortality in men in the US and the first in Puerto Rican men. PCa is a complex disease in which multiple factors may increase the risk of its development, including age, family history of PCa, ethnicity (African ancestry), obesity, hormones and certain genetic conditions (e.g., Lynch syndrome and *BRCA1* and *BRCA2* mutations) [1]. Black men in the US and Caribbean have the highest PCa incidence rates in the world [1]. According to a study by Chinea et al. (2017), Hispanic/Latino (H/L) men have a higher prostate-cancer-specific mortality (PCSM) rate when compared with Non-Hispanic Whites (NHW) in the US [2]. Moreover, Puerto Ricans (PR) had significantly a higher PCSM rate than NHW and non-Hispanic Blacks (NHB), and the highest mortality among Hispanic subgroups. The main contributors to this increased mortality in the PR H/L population are still unknown.

Dysregulation of at least three DNA repair pathways, nucleotide excision repair (NER), homologous recombination repair (HRR) and mismatch repair (MMR), has been associated with the complex carcinogenesis process in PCa development [3,4,5,6,7,8,9,10,11]. Alterations in DNA repair genes involved in HRR and MMR are among the most commonly reported in prostate tumors [12,13,14]. The identification of these alterations has provided personalized medicine options for PCa treatment, including the recent approval of PARP-1 inhibitors [15]. Although a significant proportion of prostate tumors harbor DNA damage repair (DDR) deficiencies [11], little is known regarding the DNA repair capacity (DRC) in lymphocytes from PCa patients.

DRC can be defined as the ability of a cell to repair DNA damage, which has been associated with the risks of cancer, neurodegenerative disease, inflammatory disorders and aging [16,17]. Evidence exists that DRC is an important factor contributing to the inter-individual variability in response to carcinogens and cancer susceptibility in the general population [17,18]. Epidemiological studies using functional repair assays in lymphocytes have demonstrated that DRC varies greatly among individuals and that having a low DRC level is a risk factor for the development of several types of cancer [5,19,20,21,22,23,24,25,26,27,28,29].

The only published study that has evaluated DRC levels in lymphocytes of PCa patients was performed by Hu et al. (2004) [5]. Their results show that deficient DRC levels measured through the NER pathway using the host cell reactivation (HCR) assay in lymphocytes are associated with increased PCa risk in NHW [5]. Currently, no published data are available regarding DRC levels in PR H/L PCa patients or for any other H/L subgroup. 

Decordier et al. (2010) reviewed and compared various methodologies utilized for evaluating DRC phenotypes phenotyping for DRC. Traditionally, the HCR assay with a luciferase reporter gene has been widely used to conduct large-scale population studies for different types of cancer [5,21,22,25,27,30,31]. Despite the widespread applications of the HCR assay, this technology is costly and labor-intensive, and has a limited capacity in terms of the volume of samples processed. A promising new tool with which to study DNA damage and repair, the CometChip (R&D Systems, Minneapolis, MN, USA), was developed during the last decade [32,33,34,35]. The CometChip is a high throughput technology that allows, due to its 96-well format, for the assessment of a large number of samples simultaneously with excellent reproducibility [36]. Several studies have described the potential applications and benefits of the CometChip when compared with the traditional comet assay, since it reduces experimental noise and comet-to-comet variance, and improves reproducibility [32,35,36]. Although it has been used to measure DNA damage, Ngo et al. (2021) reported that the CometChip can distinguish between DNA repair kinetics among individuals, highlighting its potential applications for future epidemiological and clinical studies [37]. Pursuant to this finding, our study represents the first report on the use of the CometChip to measure DRC levels in clinical samples, specifically from PCa patients.

The aim of this study was to evaluate variations in DRC levels PR H/L men with and without PCa and also to evaluate any relationship between DRC and prostate tumor aggressiveness. We also examined whether age, prostate-specific antigen (PSA) levels or anthropometric measures at the time of diagnosis influenced the DRC levels of the study participants. As a secondary aim, we evaluated the CometChip as a phenotypic tool to assess DRC values in human lymphocytes and to explore its potential clinical value. This initial effort consisted of 55 samples collected as part of an ongoing case-control clinic-based study. We hypothesized that variations in DRC would be detected between men with and without PCa, and that this trend would be reflected after stratifying by tumor aggressiveness. 

## 2. Materials and Methods

Use of Human Subjects and Institutional Review Board (IRB). This study was approved by the IRB of Ponce Health Sciences University/Ponce Research Institute (PHSU/PRI) prior to initiation (IRB number 2101051235R001). PRI has a consortium agreement with St. Luke’s Hospital (Ponce, PR) where the recruitment sites are located: Advance Urology and Laparoscopic Center and UroCentro del Sur. Written informed consent from all study participants was obtained by the study nurse or physicians prior to blood sample collection. Clinical and epidemiological data were abstracted from the study participants’ electronic medical records.

Study Population. Controls (men without PCa) and pre-operative PCa cases were recruited for this study. The inclusion criteria for controls were men ≥ 45 years of age, with normal results from the digital rectal exam (DRE), and normal PSA (prostate-specific antigen) levels (<4 ng/mL). Cases were PCa patients with pathologically confirmed primary PCa. Blood collection was performed at the time of diagnosis, before beginning chemotherapy or radiation.

Blood Collection. Blood extraction was completed by the recruitment sites’ nurses. Peripheral blood lymphocytes were isolated from blood samples (6 mL) using BD Vacutainer™ Glass Mononuclear Cell Preparation Tubes (CPT). For storage, the obtained lymphocytes were suspended in 2 mL of freezing media containing 10% dimethyl sulfoxide (DMSO), 40% RPMI 1640 medium, 50% FBS and 1% antibiotic/antimycotic. Aliquots were stored in a −80 °C freezer for 1–3 weeks. The lymphocytes were then thawed in batches of five samples to perform the DRC measurements using the CometChip (R&D Systems).

Cell lines. In each DRC measurement experiment, three commercial cell lines were included as internal controls. Cell lines were purchased from Coriell Institute for Medical Research (Camden, NJ, USA). The GM08925 cell line was included as a model for normal DRC. GM02246 and GM02253 cell lines were included as models of medium and low DRC, since they have knockdowns in *XPC* and *XPD*, respectively. Lymphocytes and cell lines were grown in 88% RPMI-1640, 10% fetal bovine serum (FBS), 1% L-glutamine, 1% antibiotic/antimycotic and phytohemagglutinin. All cells were grown at 37 °C in a humidified incubator containing 5% CO_2_.

DNA repair capacity (DRC) measurements. The DRC measurements were performed using the CometChip (R&D Systems, Minneapolis, MN, USA). This 96-well plate assay allows measurements of DRC levels with high reproducibility [37]. Briefly, primary lymphocytes isolated from study participants were irradiated with 20 J/m^2^ ultraviolet C (UVC) light, a DNA repair inducer which preferentially activates the NER pathway. Co-treatment with 15 μM aphidicolin C (APC) for 30 min was used to allow for the accumulation of repair incisions in lymphocytes. After allowing 2 h for repair to occur, the lymphocytes were loaded on the Chip coated with low-temperature melting agarose and lysed following the manufacturer’s instructions. After lysis, alkaline electrophoresis (200 mM NaOH/1 mM EDTA/0.1% Triton X-100) was performed and the chip containing the nuclei was stained with YOYO-1 (Invitrogen, Waltham, MA, USA). Ethyl methanesulfonate (EMS) was used as a positive control at a concentration of 12 mM for 4 h. Several images were acquired for each sample to capture 50 comets per sample using the EVOS M7000 (Invitrogen, Waltham, MA, USA ). Images were uploaded to Comet Analysis Software (R&D Systems, Minneapolis, MN, USA) for analysis of the percentage of DNA in the tail; this is the parameter used for the assessment of single-strand DNA damage. All DRC level measurements were performed in triplicate for each study participant. Calculations for the DRC levels were performed using the data obtained on the percentages of DNA in the tails of the samples with the different treatments and the equation presented in the work of Vande Loock et al. (2010) [38].

DRC = %TD (APC + UVC) − %TD (UVC) − %TD (APC)], where TD is tail density.

Statistical analysis. Analysis of variance was used to assess differences in DRC values of the three cells lines, followed by a post hoc test for multiple comparisons. Distribution of epidemiological and clinicopathological variables was analyzed using contingency tables and Fisher’s or Chi-squared (X^2^) tests. Non-parametric tests (Mann–Whitney U or Kruskal–Wallis tests) were used to assess the statistical significance of the mean differences from independent samples while accounting for non-normally distributed variables, such as DRC. Analysis of covariance was performed to assess whether age, BMI or PSA levels contributed to the variance observed in DRC values. Significance levels were established using a *p*-value cutoff of 0.05 based on a two-tail test for the proportions and mean comparisons. The Bonferroni correction was used to assess mean differences in DRC values after adjusting for age, BMI, and PSA levels. The data were analyzed using SPSS 25.0 software (Chicago, IL, USA), and Graphpad Prism 6 was used for graphical presentation.

## 3. Results

### 3.1. Epidemiological and Clinicopathological Variables

PCa cases were generally men over 55 years of age (61.0%) with body mass indexes (BMI) over 25 kg/m^2^ (84.6%) (Table 1). Regarding comorbidities, most of the PCa cases suffered from hypertension (53.8%), but the frequency of diabetes (22%) and other urological conditions (14.6%) was low. Most of the cases reported consuming alcohol (60.5%) occasionally, and very few reported smoking (26.8%). A low frequency of caffeine consumption was reported for this group (40%). Regarding the controls, these were equally distributed across the age stratifications. Similar to the PCa cases, most of the controls had a BMI over 25 kg/m^2^. Similar to the PCa cases, most of the participants in the control group suffered from hypertension (57.1%), and the frequency of urological conditions was low (14.3%). Similarly to the PCa cases, the men in the control group reported consuming alcohol (50%) occasionally. Most of the controls reported consuming more than two cups of coffee daily. Additional variables, such as family history of cancer, were also evaluated; however, no significant differences were observed between groups (*p* > 0.05).

### 3.2. DNA Repair Capacity in Prostate Cancer Cases and Controls

**Figure 1:**
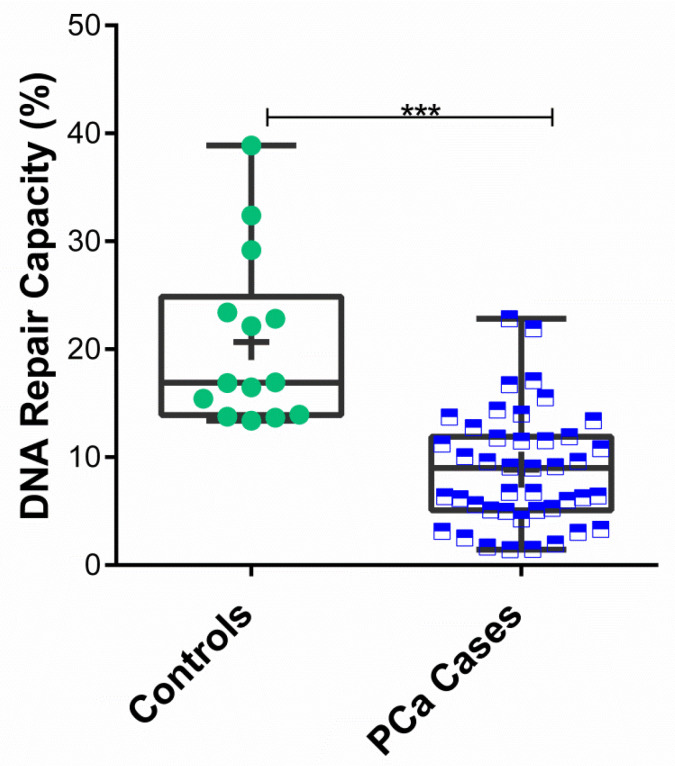
DNA repair capacity levels in prostate cancer cases and controls measured in terms of NER pathway. Sample distributions using the DRC values for PCa cases (*n* = 41) and controls (*n* = 14). Each box and whiskers represent the median and range values for a study group. Dots and squares represent the individual DRC values for PCa cases (green circles) and controls (blue squares). Mean DRC value for each group is represented with a plus (+) sign. Asterisk (***) represents significant results based on a Mann–Whitney U test, *p* < 0.001.

In order to assess variations in DRC through the NER pathway among study participants, the CometChip assay was used. Through the use of UVC light, a known NER pathway inducer, the capacity to repair DNA damage through this pathway was evaluated. A total of 55 participants were included in this analysis, including PCa cases (*n* = 41) and controls (*n* = 14) (Table S1). The mean DRC value for the control group was 20.66% (±7.96%), whereas the mean DRC for the PCa cases was 8.41% (±4.88%). To assess differences in DRC levels between cases and controls, the Mann–Whitney U test was performed. Significant differences were found when comparing the average DRC levels between cases and controls (*p* < 0.001) (Figure 1).

### 3.3. Clinicopathological Characteristics of Prostate Cancer Patients

PCa cases were stratified into indolent and aggressive groups based on the Gleason score obtained from the pathology reports. Overall, PCa cases with indolent tumors had a mean age of 59.5 years (Table 2). Most of the participants in this group had tumors with Gleason scores of seven (3 + 4), corresponding to Grade Group 2 (58.8%) and the pathological stage of pT2 pN0. Most of these patients had PSA levels above 4 ng/mL (70.6%) at the time of diagnosis. All of the participants included in the indolent group were treatment-naïve at the time of recruitment. PCa cases with aggressive tumors were older than patients in the indolent group; their mean age was 66 years (*p* = 0.04). Most of the men in this group had tumors with Gleason scores of 8–9 (65.2%), corresponding to Grade Groups 4 and 5. Most of these patients had not undergone prostatectomy and had PSA levels above 4 ng/mL (87.0%). Although some of the patients in this group received androgen deprivation therapy; most of the participants had not received treatment at the time of recruitment. Most of the patients in the indolent group had undergone radical prostatectomy. In contrast, most of the patients with aggressive tumors had not (*p* = 0.02).

### 3.4. DNA Repair Capacity in Aggressive and Indolent Prostate Cancer

**Figure 2:**
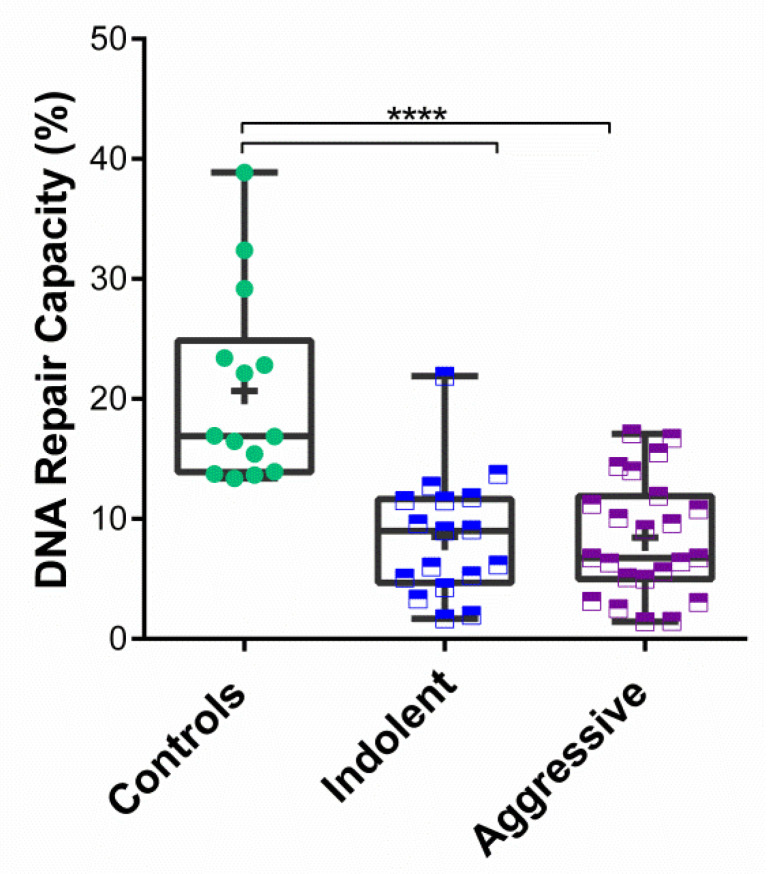
DNA repair capacity in prostate cancer patients with indolent and aggressive tumors. Based on their Gleason scores, the tumors from PCa cases were stratified into indolent (*n* = 17) and aggressive (*n* = 23). Symbols represent individual DRC values. Mean DRC value for each group is represented with a plus (+) sign. Asterisk (****) denotes statistical significance (*p* = 0.001, Kruskal–Wallis test).

To further explore the differences in DRC within the PCa cases group, stratification into aggressive and indolent PCa was performed. The indolent group included PCa cases with Gleason scores of 6 and 7 (3 + 4). The aggressive group included patients with Gleason scores of 7 (4 + 3) and higher. A total of 17 PCa cases were classified as indolent, and 23 cases were included on the aggressive group (Table S2). The mean DRC for the indolent PCa cases was 8.50% (±5.14%); for the aggressive group, the mean DRC was 8.43% (±4.88%) (Figure 2). As previously mentioned, the mean DRC for the control group was 20.66% (±7.96%). Significant differences were observed when comparing the controls with the indolent group or the aggressive group (*p* < 0.0001); however, no significant differences were detected when the PCa groups were compared to each other.

### 3.5. DRC Levels in Study Groups after Age, BMI, and PSA Level Adjustments

In order to understand whether the skewed distribution of DRC was explained by other biological factors, a general linear model analysis was performed (Table 3). In this analysis, several continuous variables were considered, including age, BMI, and PSA levels at the time of diagnosis or sample collection. The adjusted mean DRC value was 20.55% (±1.60%) for the control group, a decrease of 0.11% after covariates were considered. As for the cases, the adjusted mean DRC value was 8.45% (±0.89%), compared to 8.41% (±4.88%) obtained from the crude results. No significant contributions were detected from the cofactors in the linear model. The covariance model shows that age (*p* = 0.84), BMI (*p* = 0.50), and PSA levels (*p* = 0.27) are not statistically significant factors in the model. Although the adjusted mean DRC values slightly vary for both groups (cases and controls), the differences in DRC are still significant after the Bonferroni correction. As for the tumor aggressiveness, the linear model shows variability between the crude and estimated DRC values. The stratum of cases with aggressive tumors has an estimated DRC value of 9.28% (±1.23%), and the indolent stratum’s value is 7.86% (±1.04%). Similarly to the case–control model, the age (*p* = 0.32), BMI (*p* = 0.93), and PSA levels (*p* = 0.95) had no statistically significant impact on the model. No significant differences were detected after comparing the estimated marginal means of the tumor aggressiveness stratification (*p* = 0.40).

### 3.6. Detection of Varying DRC Levels Using the CometChip

**Figure 3:**
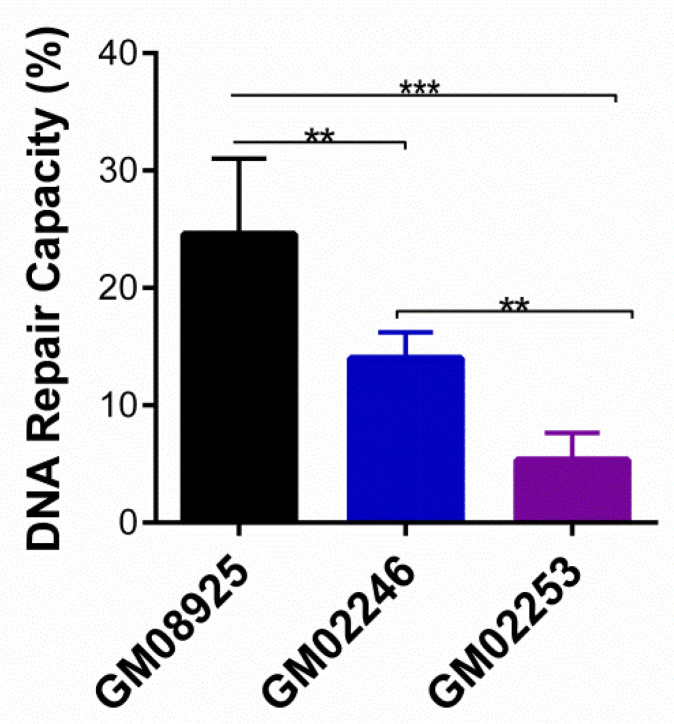
Assessment of the DNA repair capacity of human lymphocyte cell lines using the CometChip. Each bar represents the mean ± SD of three independent experiments. Asterisks denote statistical significance: (**) *p* < 0.01 and (***) *p* < 0.001.

In order to determine whether our method was able to detect varying DRC levels using the CometChip, three commercially available cell lines with different DRC levels were used as internal controls. Three Epstein–Barr virus-immortalized human lymphoblastoid cell lines obtained from Coriell Institute for Medical Research (Camden, NJ, USA) were used: (a) GM08925 was derived from a 48-year-old healthy Caucasian female; (b) GM02246 was from a 30-year-old Caucasian female with xeroderma pigmentosum complementation group C; and (c) GM02253 was from a 14-year-old Black/African American male with xeroderma pigmentosum complementation group D. We have routinely used these three cell lines for over 20 years and have established their variability in DRC levels with both the HCR assay and the CometChip. As can be observed in Figure 3, the mean DRC value for the GM08925 cell lines was 24.59% (±6.42%). As for the GM02246 and GM02253 cell lines, the mean DRC values were 14.01% (±2.20%) and 5.37 (±2.29%), respectively. Significant differences were observed when comparing the GM08925 cell lines with the GM02246 (*p* < 0.01) and GM02253 (*p* < 0.001) cell lines. Additionally, significant differences were detected when comparing GM02246 and GM02253 (*p* < 0.01). The DRC values obtained for each cell line resembled the expected results due to their genetic profiles: the highest value was detected for the GM08925 cell line, which resembles a normal DRC. Additionally, for GM02246 and GM02253, the varying levels were expected due to their genetic alterations in *XPC* and *XPD*, respectively. Therefore, our results demonstrated that the proposed method for DNA repair measurement, along with the use of the CometChip, allows for the detection of varying DRC levels in established cell lines and clinical samples.

## 4. Discussion

Prostate cancer is a complex disease, and DNA repair has been proven to play an important role both in the complex carcinogenesis process of PCa and in the biology of these tumors [3,4,5,6,7,8,9,10,11]. Although several studies have highlighted the importance of understanding the alterations in different DNA repair pathways in tumors, little is known regarding the functionality of DNA repair pathways in PCa patients. Moreover, the technology used to perform this phenotypic measure of DNA repair has also hindered our understanding and the application of the individual’s DRC in disease development and as a potential tool for patient stratification or improving diagnosis. Therefore, our study serves a dual purpose: (1) to establish the variability of DRC in PCa patients and controls in a cohort of PR H/L men and (2) to present an additional method with which to assess DRC levels in a high throughput format that can potentially allow the field of DNA repair to continue moving forward with expanding the applications of DRC levels to a clinical setting.

Although the most commonly altered DNA repair pathways in prostate tumors are MMR and HRR, NER has also been linked to PCa risk in genetic studies [4,39,40,41]. NER is a very versatile pathway and is the major pathway for repairing a variety of bulky DNA lesions (adducts), such as those induced by crosslinking agents and base-damaging carcinogens [42,43]. Additionally, NER can repair helix-distorting DNA lesions generated by environmental mutagens, such as UV irradiation [44,45]. Although the preferred pathway for repairing UV-induced DNA damage is NER, recent studies have shown a non-canonical mechanism leading to the activation of the ATM pathway in noncycling cells after UV irradiation [46,47]. Considered a “generalist” of DNA repair pathways, NER works in multiple capacities, particularly when other repair pathways exhibit reduced functionality [48].

Our main findings show that DRC, measured through the NER pathway, is reduced in men with PCa when compared to controls. Our results are similar to the findings presented by Hu et al. (2004) using the HCR assay and are consistent with the results obtained for other cancer types [21,22,27,31]. However, our study is the first to report decreased DRC in H/L PCa patients. This is extremely relevant, since H/L men have higher PCSM when compared to NHW [2]. Population studies in H/L men with PCa are very scarce in the literature and are currently underrepresented even in large genomic studies. Being that they are the second fastest growing minority in the US, our findings in this population are very relevant.

In order to further evaluate a potential relationship between DRC and tumor aggressiveness, we performed comparisons between PCa patients with aggressive and indolent tumors. Although our findings were not statistically significant, we observed a trend where PCa cases with indolent tumors had a slightly higher median DRC than participants with aggressive tumors. Interestingly, the covariance analyses showed that regardless of tumor aggressiveness, age was the major contributor in the linear model. This effect could have been due to the difference in the mean age between groups: PCa patients with indolent tumors were younger than men with aggressive tumors. Through this analysis, the trend observed in the crude results was further highlighted due to the reduced variation in the mean DRC values when age was considered. Since our method utilizes lymphocytes as surrogate markers for the individual’s DRC, this finding can provide us with additional information regarding the potential role of DRC in the development of aggressive disease, which warrants additional experimentation.

Our results also show that with our experimental setup, along with the high throughput capacity of the CometChip, it is possible to detect varying levels of DRC in clinical samples. The addition of the commercial cell lines as internal controls for the assay provides a robust setup for reproducibility. Moreover, our experimental setup provides for additional robustness, since each experimental condition is analyzed in triplicate for every clinical sample, and 50 comets are evaluated for each condition. When compared to the standard HCR assay, our method is more cost-effective, less labor intensive, and could be adapted to measure multiple DNA repair pathways. Through this study, we provide the first evidence of the applicability of the CometChip as a phenotypic tool to evaluate the DRC in PCa cases and controls.

## 5. Conclusions

Our study provides the first evidence regarding the reduced DRC in PR H/L men with PCa. Furthermore, it explores the relationship between DRC and tumor aggressiveness. Moreover, it demonstrates the applicability of the CometChip to assess DRC in clinical samples. The outcomes of this study could represent an innovative step in the development of a blood-based screening test for PCa based on DRC levels. Using a blood-based assay to measure DRC levels has several advantages: (a) changes in DRC levels can be detected in the presence or absence of a tumor, and (b) based on previous experience with breast cancer, it may (with larger sample size) provide a quantitative measure of an individual’s DRC levels and PCa risk. Future studies are warranted to evaluate DRC levels as a potential tool for early detection and also as a prognostic tool for more aggressive disease.

## Figures and Tables

**Table 1:** Epidemiological characteristics of the study population of men with and without prostate cancer.

Variables	Controls PCa *n* = 14	PCa Patients *n* = 41	*p*-Value
Age			0.41
<55	7 (50.0)	15 (36.6)	
≥55	7 (50.0)	25 (61.0)	
Missing	0 (0.00)	1 (2.43)	
BMI			0.08
<25 kg/m^2^	4 (28.6)	5 (12.8)	
≥25 kg/m^2^	7 (50.0)	33 (84.6)	
Missing	3 (21.4)	1 (2.56)	
Family history of cancer			1.00
Yes	7 (50.0)	19 (46.3)	
No	6 (42.9)	19 (46.3)	
Missing	1 (7.1)	3 (7.32)	
Hypertension			0.76
Yes	8 (57.1)	21 (53.8)	
No	5 (35.7)	17 (43.6)	
Missing	1 (7.1)	1 (2.56)	
Diabetes			0.04
Yes	7 (50.0)	9 (22.0)	
No	6 (42.9)	29 (70.7)	
Missing	1 (7.1)	3 (7.32)	
Urological conditions (not PCa)			1.00
Yes	2 (14.3)	6 (14.6)	
No	11 (78.6)	32 (78.0)	
Missing	1 (7.1)	3 (7.32)	
Alcohol consumption			0.82
Yes	7 (50.0)	23 (60.5)	
No	6 (42.9)	15 (39.5)	
Missing	1 (7.1)	0 (0.00)	
Frequency (alcohol consumption)			1.00
Occasionally	6 (85.7)	21 (91.3)	
Daily	1 (14.3)	2 (8.70)	
Smoking			0.25
Yes	6 (42.9)	11 (26.8)	
No	7 (50.0)	27 (65.9)	
Missing	1 (7.1)	3 (7.32)	
Frequency (smoking)			1.00
Former smoker	5 (83.3)	9 (81.8)	
Active smoker	1 (16.7)	2 (18.2)	
Caffeine consumption			0.11
Yes	9 (64.3)	16 (40.0)	
No	4 (28.6)	21 (52.5)	
Missing	1 (7.1)	3 (7.5)	
Frequency (caffeine consumption)			1.00
1 cup/day	3 (33.3)	7 (43.8)	
≥2 cup/day	5 (55.6)	8 (50.0)	
Missing	1 (11.1)	1 (6.25)	

*p*-value was obtained from Fisher’s exact test.

**Table 2:** Clinicopathological variables for the study group of men with prostate cancer.

Variables	Indolent PCa *n* = 17	Aggressive PCa *n* = 23	*p*-Value
Age (mean ± SD)	59.5 ± 6.3	66.0 ± 9.7	0.04
Gleason Score			<0.0001
6	7 (41.2)	0 (0.0)	
7 (3 + 4)	10 (58.8)	0 (0.0)	
7 (4 + 3)	0 (0.0)	8 (34.8)	
8–9	0 (0.0)	15 (65.2)	
Prostate Specific Antigen (PSA)			0.10
<4 ng/mL	5 (29.4)	2 (8.7)	
≥4 ng/mL	12 (70.6)	20 (87.0)	
Missing	0 (0.0)	1 (4.3)	
Prostatectomy			0.01
Yes	14 (82.4)	10 (43.5)	
No	3 (17.6)	13 (56.5)	
Grade Group			<0.0001
1	7 (41.2)	0 (0.0)	
2	10 (58.8)	0 (0.0)	
3	0 (0.0)	7 (30.4)	
4	0 (0.0)	7 (30.4)	
5	0 (0.0)	9 (39.2)	
Pathological staging			0.0008
pT2, pN0	12 (70.6)	5 (21.7)	
pT3, pN0	0 (0.0)	1 (4.3)	
pT3a, pN0	1 (5.9)	1 (4.3)	
pT3b, pN0	1 (5.9)	0 (0.0)	
pT3b, pN1	0 (0.0)	1 (4.3)	
Missing	3 (17.6)	15 (65.2)	
Androgen deprivation therapy			0.11
Yes	0 (0.0)	4 (17.4)	
No	17 (100.0)	17 (73.9)	
Missing	0 (0.0)	2 (8.70)	

*p*-value was obtained from Chi-squared and Fisher’s exact test.

**Table 3:** DNA repair capacity covariance analyses using age, BMI, and PSA levels.

Descriptive Statistics	Controls	PCa Cases	Pairwise Comparisons (*p*-Value)	Indolent PCa Cases	Aggressive PCa Cases	Pairwise Comparisons (*p*-Value)
Number of subjects	14	41	-	17	23	-
Dispersion Analysis						
Minimum	13.37	1.44	-	1.69	1.44	-
25% Percentile	13.90	5.04	-	4.68	4.99	-
Median	16.90	6.74	-	9.01	6.74	-
75% Percentile	24.86	11.65	-	11.65	11.91	-
Maximum	38.88	21.90	-	21.90	17.07	-
Analysis of covariance						
Mean	20.66 (7.96)	8.41 (4.88)	<0.0001	8.51 (5.14)	8.43 (4.88)	0.40
Estimated Mean ^a,b^	20.55 (1.60) ^a^	8.45 (0.89) ^a^	<0.0001	9.28 (1.23) ^b^	7.86 (1.04) ^b^	0.40
Lower 95% CI	16.06	6.87	-	5.86	6.32	-
Upper 95% CI	25.26	9.95	-	11.15	10.54	-
Estimated Lower 95% CI	17.41	6.66	-	6.79	5.74	-
Estimated Upper 95% CI	23.69	10.23	-	11.77	9.97	-

^a^ Case–control: Covariates appearing in the model were evaluated at the following values: age = 62.13, PSA = 38.22, BMI = 27.22. ^b^ Indolent–aggressive: Covariates appearing in the model were evaluated at the following values: age = 63.25, BMI = 29.24, PSA = 51.99. A mean difference is significant at the 0.05 level (Mann–Whitney test). Adjustment for multiple comparisons: Bonferroni.

## Data Availability

The data presented in this study are available on request from the corresponding author.

## Supplementary Materials

The following supporting information can be downloaded at: https://www.mdpi.com/article/10.3390/cancers14133117/s1, Table S1: DNA repair capacity values for the study cohort including Puerto Rican men with and without prostate cancer; Table S2: DNA repair capacity values for Puerto Rican men with indolent and aggressive prostate cancer.
