# Supplementary material for: Reduced DNA Repair Capacity in Prostate Cancer Patients: A Phenotypic Approach Using the CometChip
Source: Cancers (Basel). 2022 Jun 25;14(13):3117. doi: 10.3390/cancers14133117 (PMC9264934; doi:10.3390/cancers14133117)
Supplement: Supplementary file 1 [file cancers-14-03117-s001.zip › cancers-1753282-supplementary.pdf]

Supplementary material

Reduced DNA Repair Capacity in Prostate Cancer Patients: A Phenotypic Approach Using the CometChip

Carmen Ortiz-Sánchez, Jarline Encarnación-Medina, Jong Y. Park, Natasha Moreno, Gilberto Ruiz-Deya and Jaime Matta

Table S1. DNA repair capacity values for the study cohort including Puerto Rican men with and without prostate cancer.

Study ID	Type	DRC Results (%)
CONT-010	control	16.47
CONT-013	control	23.42
CONT-014	control	16.92
CONT-015	control	29.17
CONT-016	control	38.88
CONT-017	control	15.42
CONT-018	control	32.38
CONT-020	control	13.67
CONT-021	control	16.87
CONT-022	control	13.76
CONT-023	control	22.13
CONT-024	control	13.94
CONT-025	control	22.82
CONT-027	control	13.37
PCA-001	case	16.71
PCA-003	case	11.22
PRP-002	case	3.03
PRP-005	case	6.40
PRP-006	case	11.91
PRP-008	case	2.51
PRP-009	case	5.10
PRP-012	case	6.17
PRP-026	case	9.61
PRP-027	case	10.04
PRP-039	case	6.33
PRP-046	case	10.79
PRP-063	case	1.47
PRP-080	case	1.69
PRP-081	case	9.01
PRP-100	case	6.00
PRP-128	case	1.44
PRP-129	case	9.59
PRP-130	case	6.73
PRP-131	case	11.51
PRP-140	case	14.38
PRP-143	case	5.60
PRP-158	case	15.52
PRP-159	case	6.25
PRP-160	case	13.99
PRP-161	case	6.74
PRP-163	case	3.12
PRP-165	case	17.07

PRP-166	case	1.95
PRP-168	case	11.77
PRP-169	case	13.73
PRP-170	case	5.09
PRP-171	case	4.26
PRP-172	case	9.10
PRP-174	case	9.12
PRP-175	case	4.99
PRP-176	case	12.73
PRP-177	case	11.52
PRP-178	case	3.33
PRP-179	case	21.90
PRP-180	case	5.27

Table S2. DNA repair capacity values for Puerto Rican men with indolent and aggressive prostate cancer.

Study Cohort	Type	Disease aggressiveness	DRC Results (%)
PRP-012	case	Indolent	6.17
PRP-080	case	Indolent	1.69
PRP-081	case	Indolent	9.01
PRP-168	case	Indolent	11.77
PRP-171	case	Indolent	4.26
PRP-172	case	Indolent	9.10
PRP-180	case	Indolent	5.27
PRP-100	case	Indolent	6.00
PRP-129	case	Indolent	9.59
PRP-131	case	Indolent	11.51
PRP-166	case	Indolent	1.95
PRP-169	case	Indolent	13.73
PRP-170	case	Indolent	5.09
PRP-176	case	Indolent	12.73
PRP-177	case	Indolent	11.52
PRP-178	case	Indolent	3.33
PRP-179	case	Indolent	21.90
PRP-009	case	Aggressive	5.10
PRP-039	case	Aggressive	6.33
PRP-130	case	Aggressive	6.73
PRP-140	case	Aggressive	14.38
PRP-161	case	Aggressive	6.74
PRP-165	case	Aggressive	17.07
PRP-174	case	Aggressive	9.12
PRP-175	case	Aggressive	4.99
PRP-002	case	Aggressive	3.03
PRP-063	case	Aggressive	1.47
PRP-158	case	Aggressive	15.52
PCA-001	case	Aggressive	16.71
PRP-005	case	Aggressive	6.40
PRP-006	case	Aggressive	11.91
PRP-008	case	Aggressive	2.51
PRP-027	case	Aggressive	10.04
PRP-046	case	Aggressive	10.79
PRP-128	case	Aggressive	1.44
PRP-143	case	Aggressive	5.60
PRP-163	case	Aggressive	3.12
PCA-003	case	Aggressive	11.22
PRP-026	case	Aggressive	9.61

PRP-160	case	Aggressive	13.99
